# A Tumor Volume Segmentation Algorithm Based on Radiomics Features in FDG-PET in Lung Cancer Patients, Validated Using Surgical Specimens

**DOI:** 10.3390/diagnostics14232654

**Published:** 2024-11-25

**Authors:** Lena Bundschuh, Jens Buermann, Marieta Toma, Joachim Schmidt, Glen Kristiansen, Markus Essler, Ralph Alexander Bundschuh, Vesna Prokic

**Affiliations:** 1 Klinik und Poliklinik für Nuklearmedizin, Universitätsklinikum Bonn, 53127 Bonn, Germany; markus.essler@ukb.de (M.E.); ralph.bundschuh@uni-a.de (R.A.B.); 2 Klinik und Poliklinik für Allgemein-, Viszeral-, Thorax- und Gefäßchirurgie, Universitätsklinikum Bonn, 53127 Bonn, Germany; jens.buermann@ukb.de (J.B.); joachim.schmidt@ukb.de (J.S.); 3 Institut für Pathologie, Universitätsklinikum Bonn, 53127 Bonn, Germany; marieta.toma@ukb.de (M.T.); glen.kristiansen@ukbonn.de (G.K.); 4 Nuklearmedizin, Medizinische Fakultät Augsburg, 86156 Augsburg, Germany; 5 Department of Physics, University Koblenz, 56070 Koblenz, Germany; prokic@hs-koblenz.de; 6 University of Applied Science Koblenz, RheinAhrCampus Remagen, 53424 Remagen, Germany

**Keywords:** PET/CT, radiomics, radiation therapy treatment planning, tumor volume segmentation

## Abstract

Background: Although the integration of positron emission tomography into radiation therapy treatment planning has become part of clinical routine, the best method for tumor delineation is still a matter of debate. In this study, therefore, we analyzed a novel, radiomics-feature-based algorithm in combination with histopathological workup for patients with non-small-cell lung cancer. Methods: A total of 20 patients with biopsy-proven lung cancer who underwent [^18^F]fluorodeoxyglucose positron emission/computed tomography (FDG-PET/CT) examination before tumor resection were included. Tumors were segmented in positron emission tomography (PET) data using previously reported algorithms based on three different radiomics features, as well as a threshold-based algorithm. To obtain gold-standard results, lesions were measured after resection. Pathological volumes and maximal diameters were then compared with the results of the segmentation algorithms. Results: A total of 20 lesions were analyzed. For all algorithms, segmented volumes correlated well with pathological volumes. In general, the threshold-based volumes exhibited a tendency to be smaller than the radiomics-based volumes. For all lesions, conventional threshold-based segmentation produced coefficients of variation which corresponded best with pathologically based volumes; however, for lesions larger than 3 ccm, the algorithm based on Local Entropy performed best, with a significantly better coefficient of variation (*p* = 0.0002) than the threshold-based algorithm. Conclusions: We found that, for small lesions, results obtained using conventional threshold-based segmentation compared well with pathological volumes. For lesions larger than 3 ccm, the novel algorithm based on Local Entropy performed best. These findings confirm the results of our previous phantom studies. This algorithm is therefore worthy of inclusion in future studies for further confirmation and application.

## 1. Introduction

The integration of positron emission tomography (PET) into radiation treatment planning, and its use in the evaluation of therapeutic results, are now essential elements of practice for clinicians dealing with many tumor entities [1,2,3]. In lung cancer, especially, the advantages of PET using [^18^F]fluorodeoxyglucose (FDG) have been demonstrated not only for delineation of the primary tumor, but also for the determination of which lymph nodes should be included in the treatment field [4,5,6]. PET has also been shown to be valuable in determining prognoses of radiation treatment, especially when used in combination with radiomics analysis [7].

Though PET is now increasingly widely applied in radiation therapy treatment planning, the choice of method for delineating biological target volume (BTV)—the clinical target volume based on PET images and their quantification—remains an open question which has been the subject of many discussions [8,9,10,11,12]. Methods based on fixed uptake values are now most used; however, these are greatly limited by tumor biology and PET-imaging physics. PET uptake values for FDG and other tracers vary considerably, depending on the biology and heterogeneity of the tumor [13,14]. Physical aspects of PET imaging also influence such segmentation methods as reconstruction algorithms and post-reconstruction image processing; in addition, image noise is directly affected by the length of time between tracer injection and image acquisition, and by the total amount of injected activity [15]. Moreover, in smaller lesions, at least, algorithms based on uptake values are affected by partial volume effects (PVEs), due to the limited spatial resolution and relatively high noise of the PET. PVEs are produced because voxels at the interface between the tumor and the microenvironment are only partially filled with signals originating from the actual tumor.

To overcome the limitations of algorithms based on fixed thresholds, new algorithms were introduced based on relative uptakes, e.g., 40% of the maximum uptake in a lesion [16]. Such relative-threshold-based segmentation algorithms delivered good results, and were then further improved by including the activity of the surrounding background [17]. The next step in threshold-based segmentation methods was to introduce iterative adaptions of the threshold for each lesion based on the measured source-to-background ratio of the previous iteration step. This method, suggested by Jentzen and colleagues [18] is based on curves containing the optimal threshold for a certain lesion volume and a certain level of source-to-background activity. This threshold is then applied for the next iteration step with the new lesion volume, and the newly measured signal-to-background ratio. Use of this method also partially solves the problem of PVEs. However, this method does involve one obvious disadvantage, in that the necessary curves need to be acquired beforehand, based on phantom measurements or simulations. These curves are also strongly dependent on the choice of PET scanner and the reconstruction methods used. Adapting this method for a particular clinical setting is therefore time-consuming; in addition, procedures need to be reperformed if there is a change in equipment.

Because of the limitations of threshold-based segmentation algorithms, it is important to investigate and validate other kinds of segmentation algorithm to refine and emphasize the diagnostic and prognostic capabilities of PET. Because human observers are better able to discern changes in intensity variations and heterogeneity in grayscale images compared with absolute intensity, algorithms based on quantifications of intensity changes in the images were introduced. Geets and colleagues suggested an algorithm based on watershed transformation of image data followed by a cluster analysis [19]. In analyses of phantom data, this algorithm was found to deliver performance superior to that of segmentation algorithms, based on target-to-background ratios. Similar results were reported when active contouring algorithms were used for tumor segmentation in PET data [20]. Unfortunately, and especially for small lesions, gradient-based algorithms and active contouring are of limited clinical value. Another option which may be used to improve segmentation is the inclusion of morphological imaging information, as in modern hybrid PET imaging. Especially when PET is combined with integrated magnetic resonance imaging (MRI), superior results may be obtained, as was reported for the algorithm developed by Rundo and colleagues [21]. In the present study, segmentation algorithms in PET and MRI were combined to obtain an optimal target volume which included the good spatial resolution of MRI and the superior specificity of PET. In recent years, artificial intelligence has been used to develop algorithms for tumor volume segmentation, which have delivered superior performance using a combination of morphological and functional imaging. Deep learning has shown promising results, for example, in the feasibility study conducted by Chen et al. in which prior anatomic information was included [22]. In addition, Li and colleagues reported an especially interesting use of deep-learning-based segmentation in which CT and PET information were also used [23]. However, even mature methods involving artificial intelligence or machine learning algorithms face the challenge that, in order to be applied in clinical praxis, extensive clinical validation is required for a sufficiently high number of patients in the learning group, as well as a sufficient variety of conditions and lesions. An overview over the different segmentation algorithms is given in Table 1.

Therefore, there is still a need for alternative approaches and clinical decisions which might make valuable contributions to the enlargement of the learning group. Segmentation algorithms based on texture analysis may provide additional information about the heterogeneity of the tumor, and contribute to the quantization of tumor volume. In a previous paper, we presented a segmentation algorithm based on textural features and reported promising results based on phantom data [24]. In addition, other studies have shown the importance of radiomics analysis in lung cancer medicine [25,26].

Textural features are used to describe heterogeneity; these are derived from statistics-based methods, and are based on calculations of the local features at each pixel in the image, as well as their distribution. The segmentation algorithm proposed and described in our previous study [24] was based on a region-growing algorithm. The basic idea is that the growing of the region is ongoing until the stop criterion is reached. This stop criterion is defined as a threshold of three textural parameters: Kurtosis (KU), Local Entropy (LE), and Long-Zone Emphasis (LZE). These parameters were found to be suitable in a previous analysis [27]. With regard to textural features, the tumor size may also be of importance. Brooks et al. showed that heterogeneity measures could be very sensitive to volume in the case of smaller (<45 ccm) tumors because of the limited spatial resolution of current PET scanners [28].

In general, the evaluation and validation of tumor segmentation approaches is a difficult topic. Often, segmentation algorithms are initially validated using physical phantoms or simulated phantom data with known lesion sizes. In phantom studies, even heterogeneous lesions have been shown to validate segmentation algorithms [9,29]. In other studies, segmented tumor volumes have been compared with volumes delineated in other imaging modalities as CT or MRI. However, it is a critical matter of debate whether tumor volumes detected in one imaging modality can be validated using another one. This is especially a matter of concern because, in functional imaging such as PET, other tumor features are imaged as in morphological imaging such as CT and MRI. In addition, several limitations are associated with imaging in general. In light of such problems as spatial resolution, partial volume effects, and microscopic tumor infiltration at tumor borders, amongst others, the gold-standard method for estimating tumor volume seems to be the measurement of the tumor by a pathologist after the tumor is removed, as previously reported [30,31]. However, even this method involves limitations, as changes in the tumor tissue may occur after resection. In prostate specimens, for example, a shrinkage factor ranging from 0.50 to 1.13 after fixation in formalin was reported by the authors of [32]. In fresh or fresh-frozen specimens, however, this problem does not arise in any meaningful way; these kinds of specimens should therefore be preferred for the measurement of tumor size [33].

In this study, we continued our validation of proposed tumor segmentation methods and applied our previously reported algorithm to patients who underwent FDG-PET/CT examination before total resection of a non-small-cell lung cancer tumor. The results obtained were then compared with histopathology. After resection, the sizes of lesions were measured by an experienced pathologist before any fixation was performed. To the best of our knowledge, there have been only a limited number of studies in which texture-based segmentation algorithms have been tested by comparison with phantom measurements and with histopathology.

Because threshold-based algorithms are still widely used in clinical practice, a threshold-based algorithm was applied to the PET data for comparison with the proposed algorithms.

In brief, in this paper, we describe and validate a simple segmentation algorithm which delivers better performance than conventional, threshold-based algorithms, without the above-mentioned limitations associated with machine learning and artificial intelligence. The proposed algorithm may therefore be seen as closing the gap between these two groups of algorithms.

## 2. Materials and Methods

### 2.1. Patient Population

A total of 20 patients (14 male, 6 female, mean age 69 yrs, range 58–83 yrs) were included in this analytic study. All individuals had biopsy-proven lung cancer scheduled for primary surgical resection with curative intent and pretherapeutic FDG-PET/CT for initial staging and, especially, exclusion of distant metastases. None of the patients had previously undergone surgery or radiation treatment of the lung, and none had received any systemic antitumor treatment. All patients gave written and informed consent for all imaging procedures, and all agreed that data would be retrospectively evaluated on an anonymized basis.

All procedures involving human participants were performed in accordance with the ethical standards of the institutional and/or national research committee, and also with the 1964 Helsinki Declaration and its later amendments or comparable ethical standards. Due to the retrospective character of the data analysis, the requirement for an ethical statement was waived by the institutional review board, according to the professional regulations of the medical board of the state of Nordrhein-Westfalen, Germany.

### 2.2. Imaging

Image data were acquired using a Biograph 2 PET/CT scanner (Siemens Healthineers, Erlangen, Germany). Patients fasted for at least 6 h prior to an intravenous injection of between 272 MBq and 388 MBq (mean 351 MBq) of FDG, depending on body weight. Plasma blood glucose levels before injection were between 70 mg/dL and 166 mg/dL (mean 102 mg/dL). At a timepoint between 58 min and 129 min (mean 76 min) after the injection, a low-dose CT (16 mAs, 130 kV) from skull base to mid-thigh was acquired. The PET scan was acquired over the same area, with 3 or 4 min per bed position, depending on the body weight of the patient. CT data were reconstructed in 512-by-512 matrices with 5 mm slice thickness. PET data were reconstructed in 128-by-128 matrices with 5 mm slice thickness. An attenuation-weighted ordered-subsets expectation-maximization algorithm was utilized for attenuation and scatter corrections in line with manufacturer’s instructions. A 5 mm Gaussian post-reconstruction filter was applied to the images for smoothing.

### 2.3. Target Volume Segmentation

All segmentation was performed using software developed in-house (IDL, Version 8.5, Harris Corporation, Broomfield, CO, USA). The radiomics-based tumor volume segmentation algorithms were used in context with three textural parameters, Kurtosis (KU), Local Entropy (LE), and Long Zone Emphasis (LZE), as described previously [24]. In another previous work, these parameters were shown to be able to distinguish tumoral tissue from normal lung tissue [27]. The parameters were calculated according to the following equations:

(1) $$KU=\frac{1}{N}\sum_{i=1}^{N}\left(\frac{x_{i}-X}{stdv}\right)$$

(2) $$LE=\sum_{i=1}^{M}\sum_{j=1}^{M}{M1}_{ij}\cdot lg\left(M_{ij}\right)$$

(3) $$LZE=\frac{\sum_{i=1}^{M}\sum_{j=1}^{N}{M4}_{ij}\cdot j^{2}}{\sum_{i=1}^{N}\sum_{j=1}^{M}{M4}_{ij}}$$

where *n* is the number of voxels of the volume in which the parameter needs to be calculated, *x_i_* is the *i*-th voxel of this volume, *X* is the mean voxel value in this volume, *stdv* is the standard deviation within this volume, *M*1 is the co-occurrence matrix, and *M*4 is the gray-level size zone matrix or intensity size zone matrix of this volume, as defined, e.g., in [34,35].

The algorithms were described in detail in a previous work [24]. In brief, the segmentation algorithm is based on a region-growing algorithm, the basic idea of which is that a volume is allowed to iteratively grow a until a predefined stop criterion is reached. In the present work, this stop criterion was defined as a threshold of the parameters KU, LE, and LZE. The segmentation was performed in an interactive way, starting from a volume defined by interaction of the user with the interface (the user clicked in the center of the lesion to be delineated). Finally, the segmented volume was visualized to the user for control, with volume being additionally provided, along with the maximal diameter of the lesion. For comparison, a standard segmentation algorithm based on a variable threshold of 40% of the maximum uptake was implemented as well. This algorithm was chosen because it is still widely used, especially in lung cancer [36].

### 2.4. Pathological Workup

All included patients underwent lobectomy or pneumonectomy for complete resection of the primary lung tumor. To maintain lesion orientation and location, the specimen was marked with dice directly after removal, and subsequently transferred to the Department of Pathology at the medical center of the University of Bonn. There, the tumor was measured in three axes before any further processing or fixation was carried out; this was to avoid the shrinkage effects mentioned above. Based on these values, and assuming an ellipsoid lesion shape, the tumor volume was then calculated.

### 2.5. Statistical Analysis

Volumes segmented in the pathological workup were correlated with the volumes of the four segmentation algorithms as well as maximum diameters. The Pearson correlation coefficient (r) was then calculated, and scatter plots were produced for visualization. Bland–Altman plots were created for relative differences among these data, including upper and lower levels of agreement [37,38]. In addition, coefficients of variation were determined using the root-mean-square method (COV in %); these were assessed according to Hyslop and White [39]. For comparison of different COVs, the method reported by Forkmann was used [40]. All statistical analyses were performed using MedCalc (version 22.013, MedCalc Mariakerke, Belgium). Results were presented in the form of mean value, standard deviation, and range.

## 3. Results

In the pathological workup, 23 lesions were identified in the 20 patients; all of them were diagnosed previously in the FDG-PET/CT images. One patient with a single lesion was excluded from the analysis because he was operated upon twice. In the first resection, the complete tumor was not assessed; therefore, we did not have a single pathological specimen for further workup. Two other lesions, one with a volume of 0.1 ccm (0.5 cm diameter) and another with a volume of 0.3 ccm (1.3 cm × 0.7 cm × 0.5 cm), were excluded as the number of voxels which they filled was too small for an adequate calculation of the textural features. The remaining 20 lesions had pathological volumes ranging between 0.7 ccm and 737.9 ccm (mean 59.9 ccm ± 164.9 ccm standard deviation) and maximum diameters of 11 mm to 125 mm (mean 38 mm ± 27.3 mm standard deviation).

**Figure 1:**
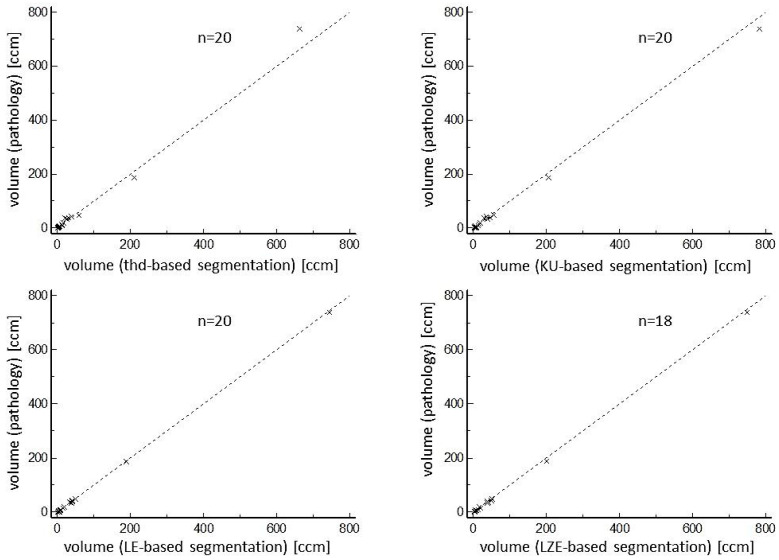
Correlations of different segmented volumes with pathological tumor volumes. Excellent correlations were found between pathological volumes and volumes of all segmentation algorithms.

**Figure 2:**
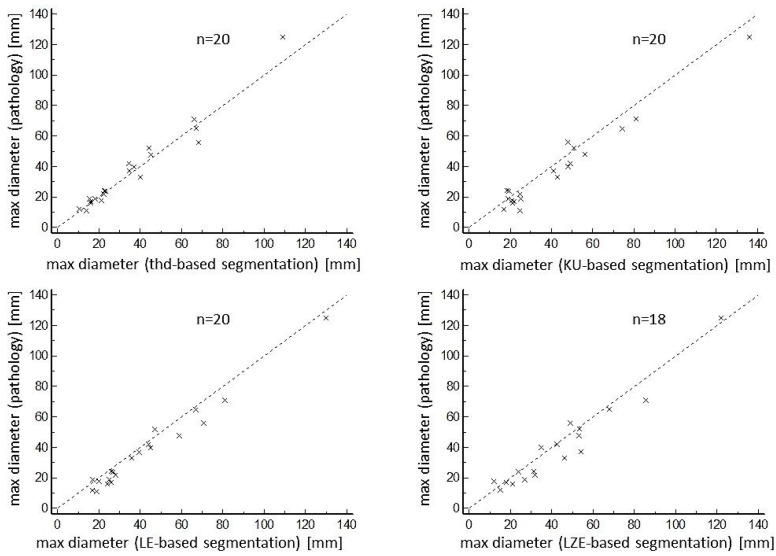
Correlations of different maximum diameters of the various segmentation algorithms with maximum diameters of the pathological workup. Excellent correlations were found between pathological maximum diameters and the maximum diameters of all segmentation algorithms.

The KU-based and LE-based algorithms, as well as the threshold-based algorithm, were able to segment all of the investigated 20 lesions. The LZE-based algorithm failed in segmentation of the two smallest lesions (0.7 ccm and 1.9 ccm). All segmented volumes correlated very well with pathological volumes as well as with maximum diameters (Figure 1 and Figure 2).

The mean segmented volume of the LE-based algorithm was 61.2 ccm ± 166.3 (mean ± standard deviation), with a range from 1.5 ccm to 745.0 ccm; this mean value was closest to the pathological volume, with a relative difference of 17.9%. The second closest value was that of the threshold-based algorithm, with a mean segmented volume of 56.2 ccm ± 150.2 ccm (range 0.6 ccm to 663.0 ccm), and a relative difference of 22.4%.

**Figure 3:**
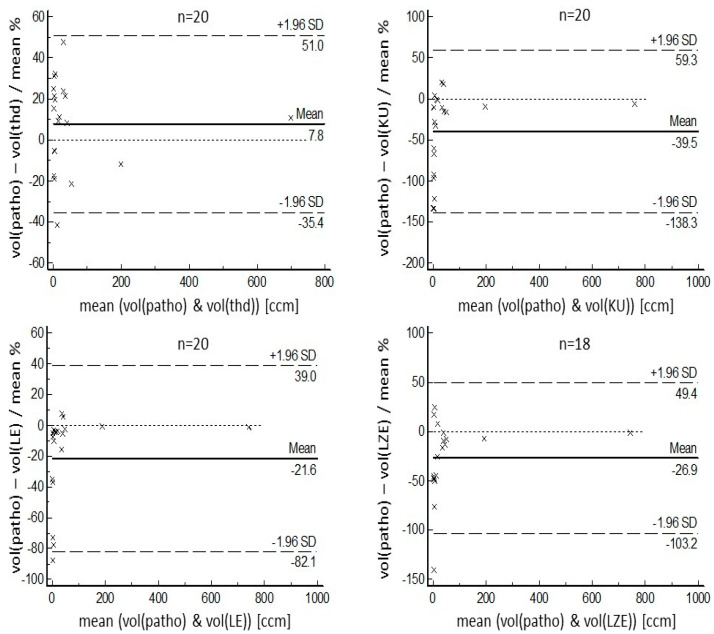
Bland–Altman plots for relative values of pathological volumes, compared with volumes segmented by the different algorithms. In agreement with other results, for larger lesions, the better the results, i.e., those with the lowest relative difference from pathological volume were found for the LE-based segmentation algorithm. For smaller lesions, threshold-based segmentation delivered better performance. KU- and LZE-based segmentation showed poorer results, but still exhibited the tendency for better agreement with larger volumes.

**Figure 4:**
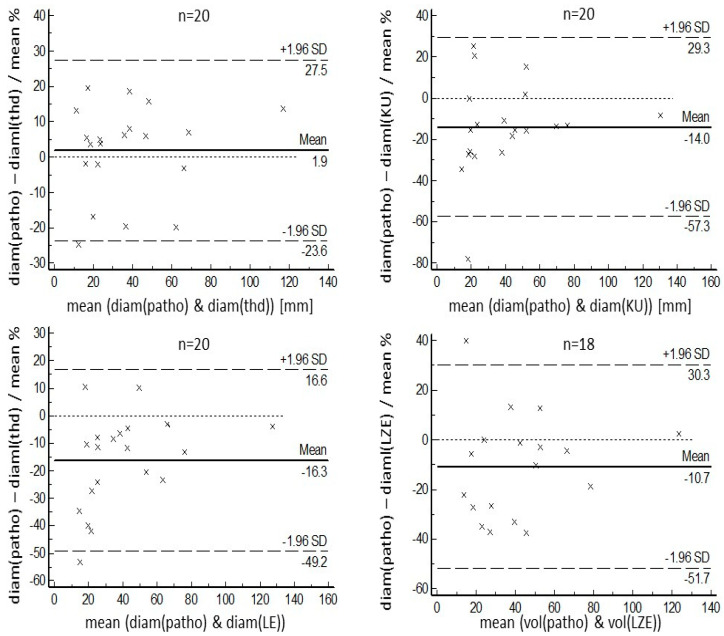
Bland–Altman plots for relative values of pathological maximum diameters, compared with the maximum diameters obtained using the different segmentation methods. In agreement with other results, for larger lesions, similar results were found for threshold-based segmentation as well as KU- and LE-based segmentation. In the smaller lesions, the best results were found for the threshold-based segmentation.

**Figure 5:**
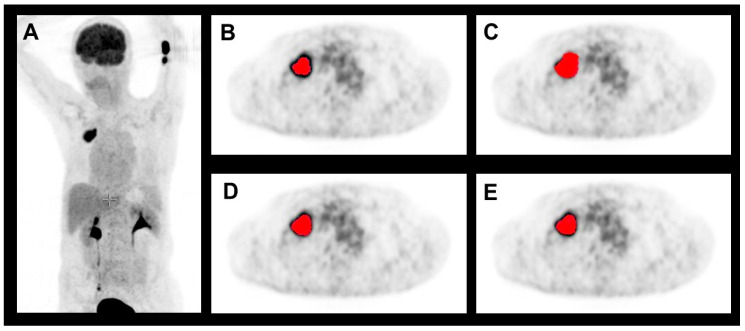
Example of a segmented lung lesion (in red) in FDG-PET for the four different segmentation algorithms with (**A**) showing the maximal intension projection of the patient. The pathological volume was measured as 38.8 ccm, which was most closely matched by the LE-based segmentation with 41.0 ccm (**D**) and the LZE-based segmentation with 39.1 ccm (**E**). As was the case for average values, in this case the threshold-based segmentation showed a volume of 31.1 ccm (**B**) which was smaller than the pathological volume, while the r-based segmentations showed a larger volume. The volume obtained using the KU-based segmentation was largest, at 49.2 ccm (**C**).

Although threshold-based volumes have a tendency to be smaller than pathological volumes, the radiomics-based volumes were larger on average. Regarding COVs for segmented volumes, threshold-based segmentation delivered the best result of 16.2%. Ranking second, but with a result which was significantly poorer statistically (*p* = 0.049), was the LE-based algorithm, with a COV of 26.2%. More details, including the results for KU-based and LZE-based segmentation, both of which performed more poorly than threshold-based and LE-based segmentation, can be found in Table 2. Regarding the maximum diameter of the segmented volumes, the threshold-based algorithm again performed best, with a mean diameter of 36 mm ± 25 mm, values which ranged from 11 mm to 109 mm, a mean relative difference of 10.8% compared with the pathological diameter, and a COV of 9.1%. The next-ranked results for COVs were 16.2% for the LZE-based segmentation, and 16.3% for the LE-based segmentation. Both these results were significantly better statistically (*p* = 0.016) than the result for threshold-based segmentation. Detailed results for maximum diameters can be found in Table 3. These results are in accordance with the Bland–Altman plots that are shown in Figure 3 and Figure 4. One patient example can be found in Figure 5.

In addition, we performed two sub-analyses: for lesions larger than 3 ccm in pathological volume (*n* = 14), and for lesions larger than 45 ccm in pathological volume (*n* = 3).

In our analysis of lesions larger than 3 ccm, we found that LE-based segmentation produced a COV of 8.3%, a result which was statistically significantly better (*p* = 0.0002) than the figure of 26.2% for all lesions. This COV of 8.3% was also significantly better (*p* = 0.018) than that of the threshold-based algorithm which did not show any significant (*p* = 0.86) difference between its COV for lesions larger than 3 ccm (16.8%) and that for all lesions (16.2%). In addition, both KU-based and LZE-based segmentation produced significantly improved results for lesions larger than 3 ccm; however, these results were still not as good as those of LE-based segmentation.

Regarding maximum diameters, we found that both LE-based and KU-based segmentation delivered improved COVs for the subgroup with lesions larger than 3 ccm. As with the results for the segmented volume, the COV for the LE-based segmentation improved significantly (*p* = 0.046) from 16.3% to 9.3% when only the group of larger lesions was taken into account. However, in terms of maximum diameter, there were no significant differences among the COVs of the threshold-based segmentation (8.6%), the KU-based segmentation (11.6%), and the LE-based segmentation (9.3%); only the LZE-based segmentation showed a significantly poorer COV result. Details about the subgroup analysis can be found in Table 1 and Table 2.

For lesions larger than 45 ccm, the LE-based segmentation showed a statistically significantly (*p* = 0.005) improved COV of 1.2%, compared with 26.2% for all lesions. The COV of the LZE-based segmentation also improved to a statistically significant degree, but was still not as good as that of the LE-based segmentation. The COV of the KU-based algorithm also improved, but without reaching statistical significance. For the three lesions larger than 45 ccm, only the LE-based algorithm had a COV which was statistically significantly better than that of threshold-based segmentation, a result most likely due to the low number of patients (*n* = 3) in this group.

For lesions larger than 45 ccm, we found that, for maximum diameter, the COVs had a tendency to improve, compared with the group of all lesions; however, a level of statistical significance was not reached, most likely due to the very limited number of lesions (*n* = 3) in this group. For maximum diameters, there were no significant differences among the COVs of the threshold-based segmentation (6.4%), the KU-based segmentation (8.5%), the LE-based segmentation (5.7%), and the LZE-based segmentation (7.9%).

## 4. Discussion

In PET imaging, the tumor data are given as a distribution of activity concentrations that correlate with the biology of the tumor, e.g., glucose metabolism in the case of FDG-PET. Radiomics analysis is a method for quantifying the heterogeneity (“texture”) of the tumor with various applications in medical imaging. In a previous paper, we reported a radiomics-features-based tumor volume segmentation in FDG-PET/CT [24]. In this previous work, the algorithm was successfully validated using phantom data, and feasibility was tested in some patient data sets. Phantom measurements are an important step in the testing of segmentation algorithms, because of the absence of biological variability that is always present in human studies. In these phantom studies, we found that segmentation based on Local Entropy provided the best results. The main objective of the present study was to quantitatively evaluate three models for radiomics-features-based tumor volume segmentation through a systematic validation using histopathology as the gold standard. Using histopathology as the gold standard should be more sophisticated than comparing volumes with other imaging modalities such as CT or MRI, which are often used in studies such as these; this is because functional imaging such as PET provides imaging information different to that of morphological imaging modalities.

In this analysis of 20 FDG-positive lesions, the algorithms based on LE and KU performed well; both were able to segment all lesions as well as the threshold-based algorithm. However, the LZE-based algorithm failed in segmentation of two small lesions with a pathological volume of less than 2 ccm. In accordance with the results of the phantom analysis, we found that all radiomics-features-based algorithms exhibit a tendency to result in volumes larger than real volumes. This tendency can be explained by the nature of the algorithm and the definition of the stop criteria at the point when the defined threshold is crossed. This may result in a serious bias in the case of small lesions. This is in accordance with our finding that, for lesions overall, the threshold-based algorithm delivered COVs which were significantly better statistically, compared with histopathological diameters and volumes. However, when lesions smaller than 3 ccm were excluded, we found that the radiomics-features-based algorithm delivered significantly improved results. Regarding comparison with pathological volumes, in the subgroup with larger lesions we found that the LE-based algorithm had a COV which was significantly better statistically than all other algorithms. In terms of other textural features, the KU-based and LZE-based algorithms had higher COVs, accordingly. These results confirm the second-order-kinetics LE-based algorithm as a robust measure of uptake heterogeneity, especially for lesions with volumes larger than 3 ccm or even larger than 45 ccm. The first-order-statistics KU-based and higher-order LZE-based models both exhibited statistically acceptable levels of agreement with histopathological volumes, but both performed more poorly than the LE-based model in this regard. Based on these results, one may speculate that these higher-order features exhibit greater instability with respect to sizes of actual lesions, and that statistical fluctuations are perhaps more critical. The performance of LZE-based segmentation seems to be especially suboptimal in this regard, most likely due to the features of the algorithm itself.

Regarding comparisons with maximum diameters, we found no statistically significant differences among the COVs of the threshold-based, the LE-based, and the KU-based algorithms; only the LZE-based algorithm showed a COV which was significantly worse statistically. Therefore, we may conclude that, for lesions larger than 3 ccm, a radiomics-features-based segmentation using Local Entropy as a parameter results in more precise segmentation of the macroscopic tumor volume in NSCLC lesions than conventional threshold-based segmentation algorithms. However, for small lesions, a threshold-based segmentation seems to be a more favorable option. These results may be included in future (semi-)automatic algorithms using the optimal algorithm based on lesion size.

The size of the tumor is very important when quantifying texture based on spatial patterns and intensity variations in PET image data. For this reason, in the present study, we analyzed tumors with volumes larger than 45 ccm. As expected, in comparisons with pathological volumes, the best results were obtained for tumors of this size. In this regard, the LE-based algorithm ranked first, with a COV which was statistically significantly better than those of all other algorithms, followed by the KU-based and LZE-based algorithms.

These results confirm the second-order-kinetics LE-based algorithm as a robust method for segmentation, especially for larger lesions. The first-order-statistics KU-based and higher-order LZE-based models both exhibited statistically acceptable levels of agreement with the histological volume, but both performed less well than the LE-based model in this regard.

The results obtained in this study from comparisons with histological volumes are in agreement with the findings of our previous phantom study [24]. We complemented our analyses of the reliability and robustness of the three textural parameters by retrospectively using a conventional measurement of lesions which was made by a pathologist when specimens arrived at the Department of Pathology. These measurements were taken before any fixation of material, so shrinkage effects should have been negligible [33]. However, more detailed measurements can be taken in a prospective setting. Moreover, for pathological volumes, we used macroscopic tumor volumes. However, if we were to consider applying the findings of the present study to radiation therapy treatment, an understanding of microscopic tumor volumes might be interesting for comparative purposes. However, this would require whole-mount pathology of the whole tumor, which was not available in this retrospective study, but may be an option in a future prospective work. For this reason, the present work should be seen as a feasibility study, and studies including more tumors of various sizes must be included to further validate this algorithm.

However, any proposed techniques which use radiomics analysis or machine learning implementations need careful validation. In light of this, another limitation of the present study, and also of the previously reported phantom study, is that all data acquisition was carried out on the same PET scanner using similar acquisition protocols, in order to focus on the analysis of the textural parameters. Any transfer of the algorithm to other systems would require a process of checking and validating to be carried out before further application. Standardization in radiomics analysis is a widely discussed topic, and various suggestions regarding such standardization have been reported [41,42]. Efforts have been made towards standardization, especially in gray-level-matrix-based radiomics, as used partly in this paper [43]. Although beyond the scope of this work, next steps might involve application of data from other PET systems as well as methods for standardization, perhaps even including (semi-)automatic steps to minimize work and further improve the relevance of the method.

In spite of these limitations, the results of our study show that the proposed segmentation algorithm based on the radiomics feature Local Entropy is a promising candidate for tumor volume segmentation in FDG-PET/CT; as such, it is worthy of further validation and use for other tumor entities.

## 5. Conclusions

The aim of the present work was to validate a novel textural-feature-based segmentation algorithm using histological volume as a gold standard, to further validate the results of our previous phantom study.

For small lung cancer lesions, conventional, threshold-based segmentation showed good results when compared with pathological volume; for lesions larger than 3 ccm, the novel algorithm based on Local Entropy performed best. This confirms and completes the results of our previous phantom studies. This novel algorithm is worthy of further validation in studies which take into account a greater variety of lesions, different scanner systems, and other tumor entities. It may also be included in future prospective studies for further validation.

## Figures and Tables

**Table 1:** Overview of literature on tumor volume segmentation mentioned in the Introduction.

	Paper	Comments
Threshold-based segmentation	[12,13,16]	Stable; fixed uptake threshold does not represent variability of glucose metabolism; problem with highly variable background depending on lesion location
Threshold-based with background adaption	[17,18]	Takes into account variations in lesion uptake and background; limited efficacy with heterogeneous tumors; strongly dependent on the system and reconstruction algorithm
Gradient-based algorithms	[19,20]	Closer to human observation, i.e., looks into changes in imaging more than intensity; use in practice limited to small lesions
Combination of functional and morphological imaging	[21]	Seems to work well in special applications but limited data available; requires high-quality anatomical data, preferably MRI
Segmentation based on machine learning	[22,23]	Promising results, but many open questions concerning standardization; large data sets necessary for training
Proposed method: Radiomics-based	Expected advantages - no larger datasets for training necessary as in AI application - represents tumor biology as tumor heterogeneity is used as measure	

**Table 2:** Head-to-head comparison of segmented volumes with gold-standard pathological volumes for all lesions (*n* = 20) and lesions larger than 3 ccm (*n* = 14). Values are given for mean value and range, along with respective mean relative difference and range, Pearson correlation coefficient (r), and coefficient of variation (COV). While all algorithms based on textural features had a mean segmented volume greater than the pathological volume, that of the threshold-based algorithm was lower. All algorithms showed very good correlation with pathological volume. Regarding COV, the best results were those of the threshold-based segmentation; however, taking into account only lesions larger than 3 ccm, segmentation based on LE showed significantly better results (*p* = 0.018).

	Mean Pathological Volume [mL]	Mean Segmented Volume [mL]	Relative Difference [%]	r	COV [%]
All lesions (*n* = 20 (18) *)					
Volume Threshold-based	59.9 (0.7–737.9)	56.2 (0.6–663.0)	22.4 (5.0–62.7)	0.997	16.2
Volume KU-based	59.9 (0.7–737.9)	64.9 (3.5–782.9)	31.2 (0.7–80.0)	0.999	44.6
Volume LE-based	59.9 (0.7–737.9)	61.2(1.5–745.0)	17.9 (0.9–60.9)	0.999	26.2
Volume LZE-based	59.9 (0.7–737.9)	70.0 (3.1–749.1)	24.8 (0.8–82.4)	0.999	32.8
Lesions > 3 ccm (*n* = 14) **					
Volume Threshold-based	84.9 (3.1–737.9)	79.5 (2.5–663.0)	22.1 (5.0–62.7)	0.997	16.8
Volume KU-based	84.9 (3.1–737.9)	90.5 (4.2–782.9)	16.2 (0.7–63.1)	0.999	20.6
Volume LE-based	84.9 (3.1–737.9)	86.1 (4.1–745.0)	7.2 (0.9–31.1)	0.999	8.3
Volume LZE-based	84.9 (3.1–737.9)	88.9 (3.3–749.1)	17.5 (0.8–39.8)	0.999	17.7
Lesions > 45 ccm (*n* = 3) **					
Volume Threshold-based	324.3 (47.7–737.9)	310.9 (59.2–663.0)	13.9 (11.1–19.3)	0.999	10.8
Volume KU-based	324.3 (47.7–737.9)	348.2 (55.9–782.9)	9.7 (5.8–14.5)	0.999	7.8
Volume LE-based	324.3 (47.7–737.9)	327.7 (49.0–745.0)	1.4 (0.9–2.5)	0.999	1.2
Volume LZE-based	324.3 (47.7–737.9)	333.9 (51.6–749.1)	5.2 (1.5–7.4)	0.999	4.3

* *n* = 20 for threshold-based, KU-based, and LE-based segmentation; *n* = 18 for LZE-based segmentation. ** For selection of the lesions, the pathological volume was used.

**Table 3:** Head-to-head comparison of diameters of segmented volumes with gold-standard diameters of pathological volumes for all lesions (*n* = 20) and lesions larger than 3 ccm (*n* = 14). Values are given for mean and range, along with respective mean relative difference and range, Pearson correlation coefficient (r), and coefficient of variation (COV). As with volumes, the diameters obtained using the threshold-based segmentation were lower than those obtained using radiomics-based segmentation. While Pearson correlations were good in all cases, the best COV results for all lesions were those of threshold-based segmentation; for lesions larger than 3 ccm, threshold-based and LE-based segmentation were both good, without significant difference (*p* = 0.78).

	Mean Pathological Maximum Diameter [mm]	Mean Segmented Maximum Diameter [mm]	Relative Difference [%]	r	COV [%]
All lesions (*n* = 20 (18) *)					
Volume Threshold-based	38 (11–125)	36 (11–109)	10.8 (1.8–22.0)	0.989	9.1
Volume KU-based	38 (11–125)	42 (17–136)	18.3 (0.0–56.0)	0.981	18.1
Volume LE-based	38 (11–125)	42 (17–130)	16.2 (3.0–42.1)	0.987	16.3
Volume LZE-based	38 (11–125)	44 (12–122)	17.2 (0.0–50.0)	0.968	16.2
Lesions > 3 ccm (*n* = 14) **					
Volume Threshold-based	47 (18–125)	46 (21–109)	10.4 (1.8–20.7)	0.973	8.6
Volume KU-based	47 (18–125)	51 (19–136)	14.9 (2.0–29.0)	0.983	11.6
Volume LE-based	47 (18–125)	51 (20–130)	10.7 (3.0–21.4)	0.987	9.3
Volume LZE-based	47 (18–125)	51 (12–122)	16.5 (0.0–50.0)	0.960	15.8
Lesions > 45 ccm (*n* = 3) **					
Volume Threshold-based	87 (65–125)	81 (66–109)	8.4 (3.1–14.7)	0.994	6.4
Volume KU-based	87 (65–125)	97 (75–136)	11.1 (8.1–12.8)	0.999	8.5
Volume LE-based	87 (65–125)	93 (67–130)	6.4 (3.0–12.4)	0.993	5.7
Volume LZE-based	87 (65–125)	92 (68–122)	8.0 (2.5–17.2)	0.972	7.9

* *n* = 20 for threshold-based, KU-based, and LE-based segmentation; *n* = 18 for LZE-based segmentation. ** For the selection of the lesions, the pathological volume was used.

## Data Availability

Because of German regulations regarding data protection, we cannot make patient data available online or send it. However, all data are available for revision on-site. Due to industrial collaboration and potential patent applications, we cannot make code available.
